# Workplace exposures associated with COVID-19: evidence from a case-control study with multiple sampling periods in England, August–October 2020

**DOI:** 10.1017/S0950268822000863

**Published:** 2022-05-12

**Authors:** Iina Hiironen, María Saavedra-Campos, Jennifer Panitz, Thomas Ma, Olisaeloka Nsonwu, Andre Charlett, Gareth J. Hughes, Isabel Oliver

**Affiliations:** 1 UK Health Security Agency, London, UK; 2 UK Health Security Agency, Leeds, UK; 3 University of Bristol, Bristol, UK

**Keywords:** Case-control study, COVID-19, meta-analysis, risk factors, SARS-CoV-2, transmission, workplace

## Abstract

We investigated risk factors associated with COVID-19 by conducting a retrospective, frequency-matched case-control study, with three sampling periods (August–October 2020). We compared cases completing routine contact tracing to asymptomatic population controls. Multivariable analyses estimated adjusted odds ratios (aORs) for non-household community settings. Meta-analyses using random effects provided pooled odds ratios (pORs). Working in healthcare (pOR 2.87; aORs 2.72, 2.81, 3.08, for study periods 1–3 respectively), social care (pOR 4.15; aORs 2.46, 5.06, 5.41, for study periods 1–3 respectively) or hospitality (pOR 2.36; aORs 2.01, 2.54, 2.63, for study periods 1–3 respectively) were associated with increased odds of being a COVID-19 case. Additionally, working in bars, pubs and restaurants, warehouse settings, construction, educational settings were significantly associated. While definitively determining where transmission occurs is impossible, we provide evidence that in certain sectors, the impact of mitigation measures may only be partial and reinforcement of measures should be considered in these settings.

## Introduction

Non-pharmaceutical interventions (NPIs) play a critical role in the COVID-19 response, and are likely to remain important interventions for infection control despite high effectiveness of vaccines [1]. Governments and public health authorities require evidence on factors associated with transmission of SARS-CoV-2 to inform control measures, including restrictions on activities. Transmission is a continuous risk primarily determined by contact patterns, behavioural, environmental and socio-economic factors [2, 3]. Some settings are likely to facilitate or amplify the risk of transmission due to a combination of behavioural and environmental factors [4]. Close-proximity contact, contact over a prolonged period of time and multiple contacts in a confined, poorly ventilated space pose the greatest risk for SARS-CoV-2 transmission [2, 5]. The opportunities for being exposed in certain settings and through certain activities have changed over time with the implementation and lifting of NPIs.

The risk of transmission of SARS-CoV-2 is highest in household settings, particularly in multioccupancy households mainly due to the high number of close contacts [6]. Certain occupations such as working in healthcare [7–9] are known to be at high risk of exposure to SARS-CoV-2. Hospitality venues including restaurants, nightclubs and bars have been reported as common exposures in large outbreaks and clusters of COVID-19 [8, 10–12]. Outbreaks have also been reported in some occupational settings including factories and warehouses [8, 13] and in educational settings [14, 15]. However, information on the role of community settings in facilitating transmission is still limited [5, 6, 8, 16].

We conducted three a case-control study in England with three sampling periods between August 2020 and October 2020 with the aim of developing improved understanding of settings and activities potentially associated with transmission of COVID-19 in England. The studies focused on non-household activities.

## Methods

### Study design and setting

We conducted a retrospective case-control study, frequency-matched for age group (18–27, 28–37, 38–47, 48–57, ≥58 years) and geographical region (East Midlands, East of England, London, South East, South West, West Midlands, Yorkshire and Humber) to ensure a representative sample of controls and to account for regional variations in restrictions to activities. The study had three sampling periods: August, September and October 2020. The study population consisted of adults (aged 18 years or over) resident in England. Cases were individuals who had tested positive for SARS-CoV-2 and completed the NHS Test and Trace (NHS T&T) contact tracing questionnaire, and who were not reported to NHS T&T as household contacts of a confirmed case. Cases were randomly sampled based on these criteria from the NHS T&T database. Controls were members of the public, registered as volunteers for a market research panel. Controls were excluded if they had tested positive for SARS-CoV-2 or reported COVID-19 symptoms in the 7 days prior to completing the survey. A power calculation was performed for the initial sampling period and used for further samples without further refinement. With a false-positive error probability of 0.05, a total sample size of 4000 subjects, a 1:1 ratio of controls to cases and a minimum important odds ratio of 2, each sampling period had a power of ≥0.9 for exposure proportions of ≥0.02 in the control group.

We introduced pragmatic plausibility thresholds for numbers of different activities reported, and excluded individuals who reported high numbers of different types of workplace exposures that appeared unrealistic, likely representing reporting errors (>1 response: emergency services, military, immigration or border force services; >2 responses: social and home care, retail, hospitality, construction and manufacturing, warehouses, food production or agriculture, transport, work-related travel; >3 responses: education, arts and education, healthcare, close contact services).

**Fig. 1.**
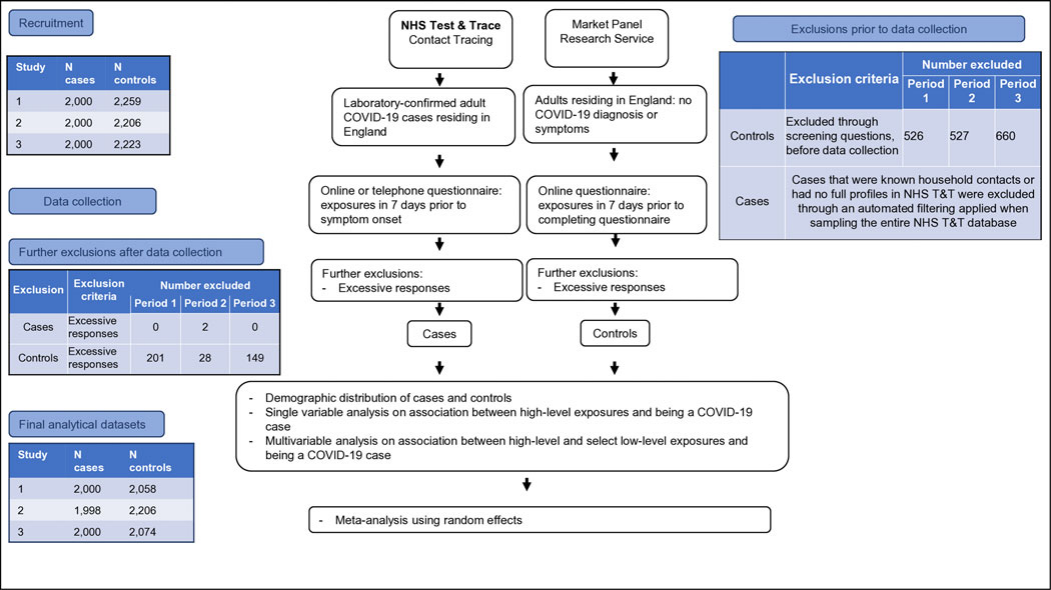
Study flow.

A different sample of controls was obtained for each study period. The sampling periods were late August 2020, late September 2020 and late October 2020, for sampling periods 1, 2 and 3, respectively. Figure 1 outlines the recruitment process and study flow.

### Data collection

Case data were collected as part of routine contact tracing, where cases provide the information either through a digital route (self-completed) or through telephone interview with a contact tracer. The contact tracing programme completion rate for cases during the three study periods was 82.7%, 76.8% and 85.0%, respectively [17].

Information on individuals' activities (exposures) was collected in a structured manner with any activity categorised by three nested levels providing increasing level of detail on activities. The highest level (Level 1) categorises the exposures to work and education activity, or leisure activities. The further information includes sectors, for example, educational, or hospitality setting. The most granular level (Level 3) of categorisation describes the workplace or the setting in more detail, for example, a pub or bar, or a secondary school. An example of this three-level structure for an exposure event associated with working in a warehouse is: Level 1 (least granular): ‘work or education’; Level 2: ‘warehouse or distribution’; Level 3 (most granular): ‘warehouse’.

Controls completed an online survey with the same exposure questions structured identically to the case questionnaire. We used the postcode of residence of the cases and controls to adjust for socio-economic deprivation using the Index for Multiple Deprivation (IMD) [18].

### Statistical analysis

Cases and controls were described according to their demographic characteristics (age, sex, ethnicity, region of residence and IMD). Single variable (logistic regression) analyses were undertaken to estimate unadjusted odds ratios (uORs) as crude measures of association between exposures (non-household activities) and outcome (confirmed COVID-19). Confidence intervals (CI) and *P* values (Wald) were calculated.

Sampling periods had a number of settings in which the number of individuals reporting ‘exposure’ was small, leading to the phenomenon of separation. We used the Firth correction [19] to obtain finite parameter estimates through penalised maximum likelihood estimation, and to reduce the biases associated with separation. All work and leisure activities on a sector level were included in the regression model, unless there was evidence of multicollinearity. Demographic variables were considered as potential confounders and were only excluded if there was evidence of multicollinearity. Multicollinearity was assessed through exploring correlation between variables in the final model using Pearson's correlation statistic. A correlation statistic >0.80 was considered as evidence of multicollinearity.

Initially a random-effects meta-analysis was used to combine the estimated odds ratios from the multivariable analyses using sector-level exposures. Further meta-analyses explored the associations of the granular level workplace activities. The sector level exposures were broken down to their most granular level (Level 3) if the sectors showed evidence of a risk association (aOR >1.5, 95% CI not including 1) in all three study periods, or a trend of a substantial increase in the observed odds over the three periods. Other exposures were included as sector-level exposures.

## Results

### Descriptive analysis

Across the three study periods, a total of 12 338 individuals were included, of which 6000 were cases. Overall, 3214 (54%) of cases self-completed contact tracing using the digital route, with limited variation between study periods (period 1: 1129 (56%); period 2: 964 (48%); period 3: 1121 (56%)). Females were overrepresented: 6926 (56%) individuals, of which 3268 (47%) were cases. Across the study periods, cases reported fewer activities (median 2, range 1–20) than controls (median 4, range 1–68). There was a greater proportion of individuals in the control group that were of white ethnicity (83%) compared to the cases (65%), although ethnicity was not recorded in 9% of case respondents. A greater proportion of cases lived in areas of low deprivation (17%) than controls (12%), although deprivation score was unknown for a large proportion of control respondents (11%). Case and control distribution was largely similar for all other demographic variables (Table 1).

**Table 1.** Demographic distribution of cases and controls by study period, England, August–October 2020

Demographic variable	Study period 1				Study period 2				Study period 3			
	Cases (*n* = 2000)		Controls (*n* = 2058)		Cases (*n* = 2000)		Controls (*n* = 2206)		Cases (*n* = 2000)		Controls (*n* = 2074)	
	*n*	%	*n*	%	*n*	%	*n*	%	*n*	%	*n*	%
Sex												
Male	870	44%	836	49%	831	42%	942	43%	891	45%	889	43%
Female	1095	55%	1214	51%	1104	55%	1262	57%	1069	53%	1182	57%
Other sex	0	0	8	0	NA	NA	2	0	NA	NA	3	0
Missing sex	35	2%	0	0	65	3	0	0	40	2%	0	0
Age group												
18–27	697	35%	694	34%	658	33%	730	33%	490	24%	505	24%
28–37	478	24%	536	26%	451	23%	493	22%	458	23%	471	23%
38–47	338	17%	349	17%	309	15%	420	19%	368	18%	411	20%
48–57	294	15%	256	12%	344	17%	237	11%	408	20%	315	15%
58+	193	10%	223	11%	238	12%	326	15%	276	14%	372	18%
Missing age	0	0	0	0	0	0	0	0	0	0	0	0
Ethnicity												
White	1180	59%	1625	79%	1264	63%	1839	83%	1426	71%	1804	87%
Mixed	75	4%	72	3%	54	3%	70	3%	50	2%	60	3%
Asian	274	14%	242	12%	241	12%	183	8%	168	8%	126	6%
Black	78	4%	87	4%	79	4%	87	4%	32	2%	63	3%
Other	223	11%	32	2%	170	8%	27	1%	132	7%	21	1%
Missing ethnicity	170	8%	0	0	192	10%	0	0	192	10%	0	0
Deprivation score												
1 (most deprived)	412	21%	457	22%	401	20%	455	21%	510	26%	557	27%
2	441	22%	462	22%	486	24%	479	22%	410	20%	420	20%
3	401	20%	322	16%	409	20%	396	18%	377	19%	326	16%
4	416	21%	315	15%	362	18%	334	15%	380	19%	302	15%
5 (least deprived)	330	16%	254	12%	342	17%	289	13%	323	16%	248	12%
Missing IMD	0	0	248	12%	0	0	253	11%	0	0	221	11%
Region												
East of England	174	9%	133	6%	109	5%	138	6%	119	6%	40	2%
East Midlands	97	5%	210	10%	190	10%	261	12%	226	11%	233	11%
London	514	26%	518	25%	500	25%	551	25%	183	9%	210	10%
North East	85	4%	118	6%	108	5%	151	7%	111	6%	286	14%
North West	269	13%	235	11%	258	13%	187	8%	507	25%	740	36%
South East	293	15%	241	12%	291	15%	243	11%	163	8%	110	5%
South West	201	10%	140	7%	209	10%	170	8%	137	7%	62	3%
West Midlands	228	11%	299	15%	201	10%	291	13%	226	11%	293	14%
Yorkshire and Humber	139	7%	164	8%	134	7%	214	10%	328	16%	100	5%

### Single variable analysis

There was statistical evidence that working in social or domiciliary care, health care or hospitality was associated with being a COVID-19 case in all three study periods (Table 2). Additionally, study periods 2 and 3 showed evidence that working in a warehouse, emergency services and close contact services were each significantly associated with being a case (Table 2). Furthermore, in these two periods, working or attending education was associated with increased odds of illness (Table 2). The most granular work settings within each sector are included in the footnotes of Tables 2 and 3.

**Table 2.** Single variable analysis results for associations between occupational exposures and becoming a case of COVID-19 by study period, England, August–October 2020

	Period 1							Period 2							Period 3						
	Cases		Controls					Cases		Controls					Cases		Controls				
	Exposed		Exposed		Odds ratio	95% CI	*P* value	Exposed		Exposed		Odds ratio	95% CI	*P* value	Exposed		Exposed		Odds ratio	95% CI	*P* value
	*n*	%	*n*	%				*n*	%	*n*	%				*n*	%	*n*	%			
Social care^a^	169	8.5	39	1.9	4.78	(3.33–6.99)	<0.001	95	4.8	21	1	4.38	(2.74–7.26)	<0.001	52	2.6	25	1.2	2.19	(1.33–3.69)	0.001
Health care^b^	199	10	103	5	2.1	(1.63–2.71)	<0.001	176	8.8	73	3.6	2.21	(1.69–2.91)	<0.001	197	9.9	75	3.6	2.91	(2.20–3.88)	<0.001
Hospitality^c^	98	4.9	54	2.6	1.91	(1.35–2.73)	<0.001	91	4.6	34	1.7	2.61	(1.76–3.96)	<0.001	86	4.3	47	2.3	1.94	(1.33–2.84)	<0.001
Close contact services^d^	33	1.7	24	1.2	1.42	(0.81–2.52)	0.19	22	1.1	8	0.4	2.51	(1.11–6.21)	0.016	38	1.9	8	0.4	5	(2.29–12.44)	<0.001
Construction^e^	77	3.9	75	3.6	1.06	(0.76–1.48)	0.73	99	5	40	2	1.85	(1.31–2.63)	<0.001	154	7.7	59	2.8	2.85	(2.08–3.94)	<0.001
Warehouse^f^	15	0.8	18	0.9	0.86	(0.40–1.81)	0.659	29	1.5	6	0.3	4.99	(2.03–14.74)	<0.001	53	2.7	4	0.2	14.09	(5.17–53.67)	<0.001
Food production and agriculture^g^	17	0.9	24	1.2	0.73	(0.37–1.41)	0.314	31	1.6	8	0.4	2.13	(1.11–4.25)	0.015	17	0.9	17	0.8	1.04	(0.50–2.17)	0.915
Retail^h^	74	3.7	116	5.6	0.64	(0.47–0.87)	0.004	68	3.4	60	2.9	0.89	(0.63–1.25)	0.479	73	3.7	74	3.6	1.02	(0.73–1.44)	0.888
Transport^i^	37	1.9	64	3.1	0.59	(0.38–0.90)	0.01	42	2.1	37	1.8	0.86	(0.56–1.32)	0.464	59	3	34	1.6	1.82	(1.17–2.88)	0.005
Emergency services^j^	21	1.1	37	1.8	0.58	(0.32–1.02)	0.045	10	0.5	7	0.3	1.46	(0.50–4.53)	0.44	25	1.3	10	0.5	2.61	(1.21–6.11)	0.008
Arts, entertainment and recreation^k^	18	0.9	41	2	0.45	(0.24–0.80)	0.004	27	1.4	30	1.5	0.74	(0.43–1.26)	0.239	30	1.5	17	0.8	1.84	(0.98–3.57)	0.042
Work related travel^l^	15	0.8	62	3	0.24	(0.13–0.43)	<0.001	21	1.1	20	1	0.79	(0.42–1.46)	0.424	13	0.7	36	1.7	0.37	(0.18–0.72)	0.001
Education^m^	35	1.8	172	8.4	0.2	(0.13–0.28)	<0.001	238	11.9	129	6.3	1.38	(1.12–1.70)	0.002	290	14.5	198	9.6	1.61	(1.32–1.96)	<0.001
Immigration^n^	1	0.1	9	0.4	0.11	(0.00–0.82)	0.013	1	0.1	3	0.2	0.34	(0.01–4.24)	0.327	0	0	1	0.1	0	(0.00–.)	0.326
Military^o^	0	0	11	0.5	0	(0.00–0.36)	0.001	11	0.6	3	0.2	3.76	(0.99–21.01)	0.029	10	0.5	3	0.1	3.47	(0.89–19.64)	0.044

a Working in social care – including care homes, domiciliary care, in-home carers and health visitors.

b Working in healthcare – including hospitals, GPs, drop-in clinics, GPs, community hospitals, ambulance services and other healthcare professions.

c Working in hospitality – including restaurants, food and drink outlets and lodging.

d Working in close contact services – including barbers, hairdressers, beauty and nail salons, make-up studios, tattoo studios, spas and wellness business, tanning salons and any other services which require close contact.

e Working in manufacturing or construction – including construction labour or office-based roles, other construction professions and manufacturing jobs in textiles, electronics, cars, furniture, pharmaceuticals, chemical plants, printing, engineering.

f Working in warehouse settings – including working in warehouse, haulage, wholesale or food distribution.

g Working in food production – including farming and agriculture, any food manufacturing and food production.

h Working in retail – working in retail stores, supermarkets, newsagents, food stores and other retail related professions.

i Working in transport – including working in underground or tram, trains, buses and logistics and storage.

j Working in emergency services – including fire services, police and other emergency services.

k Working in arts, entertainment or recreation – including music, theatre, gyms, cinema, leisure centres.

l Work-related travel – including attending conferences, door-to-door sales, home care visits and visiting clients or sites.

m Education – working only: applicable to childminder, nursery, primary school, secondary school and sixth form and working or attending applicable to university, higher education and special needs educational settings.

n Working in immigration or border force services – including office-based and people facing professions.

o Working in the military – including the Navy, the Army and the Air Force.

**Table 3.** Multivariable analysis results for associations between occupational exposures and becoming a case of COVID-19 by study period, England, August–October 2020

	Period 1				Period 2				Period 3			
Work exposure	Odds ratio*	95% CI		*P* value	Odds ratio*	95% CI		*P* value	Odds ratio*	95% CI		*P* value
Warehouse^a^	1.18	0.39	3.55	0.765	5.29	1.87	14.97	0.002	15.17	4.57	50.37	<0.001
Social care^b^	5.68	3.52	9.18	<0.001	4.94	2.85	8.56	<0.001	2.42	1.34	4.35	0.003
Hospitality^c^	2.87	1.73	4.75	<0.001	2.93	1.81	4.74	<0.001	2.14	1.34	3.42	0.001
Healthcare^d^	2.83	1.98	4.05	<0.001	2.73	1.94	3.83	<0.001	3.09	2.2	4.35	<0.001
Construction^e^	1.37	0.82	2.27	0.229	1.99	1.29	3.06	0.002	2.65	1.79	3.93	<0.001
Education^f^	0.3	0.19	0.47	<0.001	1.62	1.25	2.11	<0.001	2.03	1.59	2.6	<0.001
Transport^g^	1.07	0.57	1.99	0.838	0.78	0.47	1.3	0.348	2.13	0.91	5	0.081
Emergency services^h^	0.59	0.28	1.23	0.157	1.84	0.59	5.73	0.293	1.98	0.71	5.52	0.194
Close contact services^i^	2.92	1.11	7.68	0.03	1.11	0.4	3.04	0.843	1.24	0.83	1.86	0.293
Retail^j^	0.84	0.56	1.25	0.391	1.01	0.68	1.52	0.945	1.78	1.05	3.03	0.032
Work-related travel^k^	0.39	0.18	0.82	0.013	0.78	0.37	1.62	0.498	0.48	0.23	1	0.05
Arts, entertainment, recreation^l^	0.86	0.39	1.93	0.719	0.75	0.4	1.43	0.387	1.61	0.77	3.38	0.204
Immigration services^m^	1.34	0.07	25.6	0.845	0.3	0.03	3.52	0.337	2.6	0.1	66.58	0.564
Military^n^	0.05	0	3.45	0.162	6.53	1.16	36.81	0.033	4.8	0.86	26.89	0.075
Food production and agriculture^o^	1.2	0.41	3.54	0.742	1.84	0.85	3.97	0.121	0.9	0.36	2.24	0.814

a Working in warehouse settings – including working in warehouse, haulage, wholesale or food distribution.

b Working in social care – including care homes, domiciliary care, in-home carers and health visitors.

c Working in hospitality – including restaurants, food and drink outlets and lodging.

d Working in healthcare – including hospitals, GPs, drop-in clinics, GPs, community hospitals, ambulance services and other healthcare professions.

e Working in manufacturing or construction – including construction labour or office-based roles, other construction professions, and manufacturing jobs in textiles, electronics, cars, furniture, pharmaceuticals, chemical plants, printing, engineering.

f Education – working only: applicable to childminder, nursery, primary school, secondary school and sixth form and working or attending applicable to university, higher education and special needs educational settings.

g Working in transport – including working in underground or tram, trains, buses and logistics and storage.

h Working in emergency services – including fire services, police and other emergency services.

i Working in close contact services – including barbers, hairdressers, beauty and nail salons, make-up studios, tattoo studios, spas and wellness business, tanning salons and any other services which require close contact.

j Working in retail – working in retail stores, supermarkets, newsagents, food stores and other retail-related professions.

k Work-related travel – including attending conferences, door-to-door sales, home care visits and visiting clients or sites.

l Working in arts, entertainment or recreation – including music, theatre, gyms, cinema, leisure centres.

m Working in immigration or border force services – including office-based and people facing professions.

n Working in the military – including the Navy, the Army and the Air Force.

o Working in food production – including farming and agriculture, any food manufacturing and food production.

*Adjusted for age, sex, region, ethnicity, index of multiple deprivation and leisure activities.

We did not observe any substantial differences in uOR for point estimates obtained for level 2 workplace exposure groups using only cases completing contact tracing by the two different routes; across all three study periods: median difference = 0.29 (interquartile range 0.12–0.67) (full results not shown). Although some changes in statistical significance were seen when comparing these uOR, there was no change in the direction of the association (i.e. odds ratios both <1 or >1).

### Multivariable analysis for occupational exposures

All multivariable analyses were adjusted for age, sex, ethnicity, socio-economic deprivation (IMD), geographical region and leisure activities. All three study periods showed an association between working in healthcare, social care or hospitality, and becoming a COVID-19 case (Table 3). Working in warehouse settings, construction and manufacturing and working or attending educational settings were all associated with increased odds of infection in the second and third study periods, with the strength of the observed association increasing substantially over the three study periods (Table 3).

### Meta-analysis of sector-level work-related exposures

**Fig. 2.**
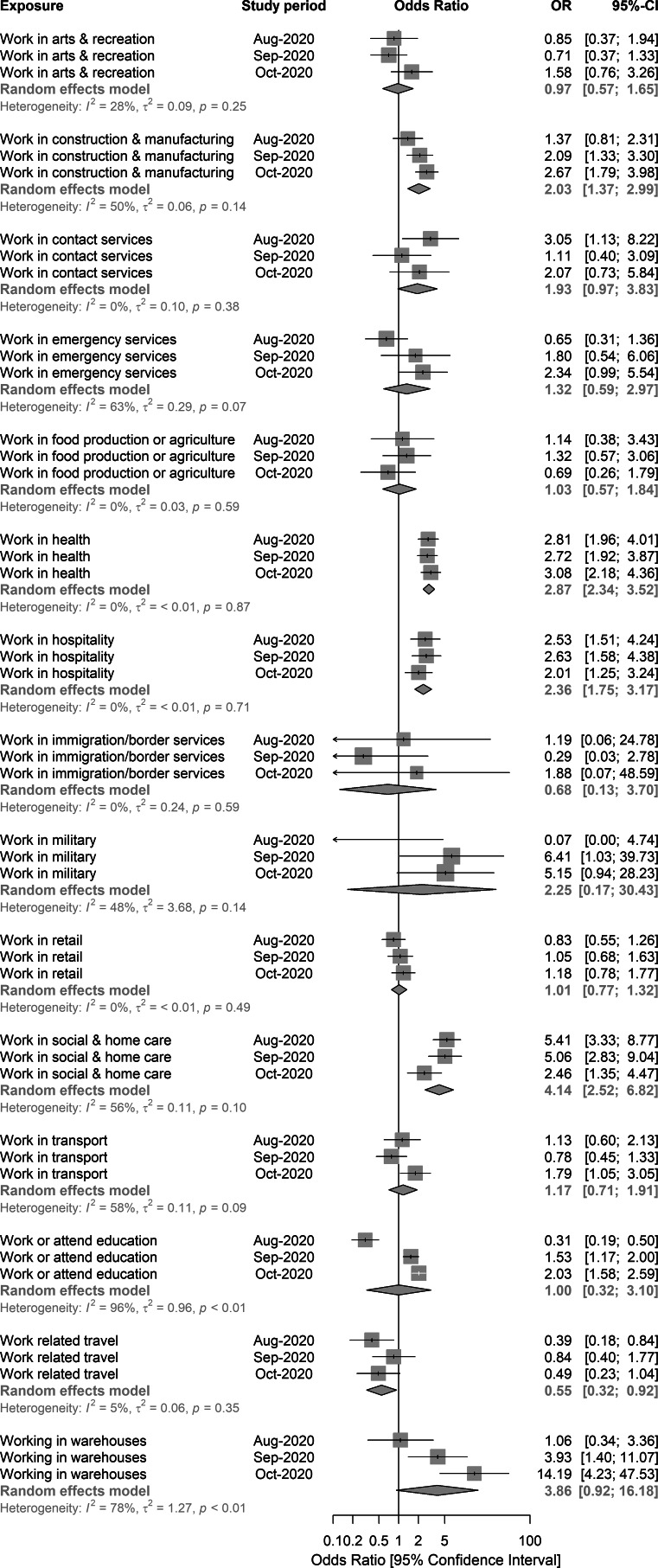
Random-effects meta-analysis of the three case-control study periods – associations between grouped, sector-level workplace exposures, England, August–October 2020.

The estimated odds ratios for work-related exposures used in the meta-analyses were adjusted for age, sex, ethnicity, IMD, geographical region and leisure activities. There was evidence that working in healthcare (pooled odds ratio (pOR) 2.87; aORs 2.81, 2.72, 3.08, for studies 1–3 respectively), social care (pOR 4.14; aORs 5.41, 5.06, 2.46, for studies 1–3 respectively) or hospitality (pOR 2.36; aORs 2.53, 2.63, 2.01, for study periods 1–3 respectively) were associated with increased odds of being a COVID-19 case. There was also evidence that working in a warehouse setting was associated with increased odds (pOR 3.86; aORs 1.06, 3.93, 14.19, for study periods 1–3 respectively). The aORs for the association between warehouses and the odds of infection showed a substantial increase over the three study periods, also indicated by the evidence of heterogeneity between the observed aORs (*I*^2^ = 78%, *P* < 0.01). A similar pattern was also observed in construction (pOR 2.03; aORs 1.37, 2.09, 2.67, for studies 1–3 respectively), and in educational settings (pOR 1.00; aORs 0.31, 1.53, 2.03, for studies 1–3 respectively) (Fig. 2).

### Meta-analysis of granular level workplace-related exposures

**Fig. 3.**
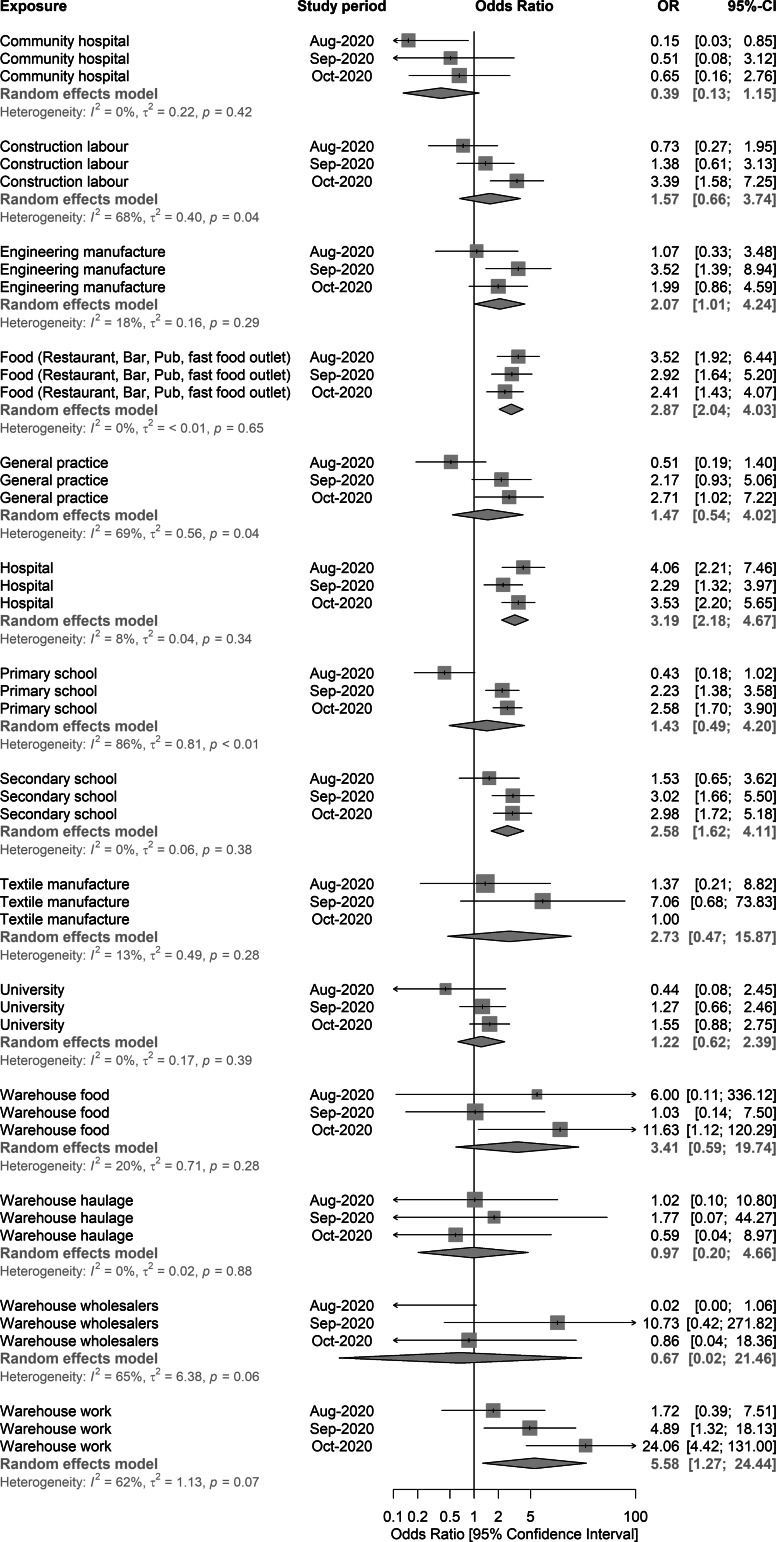
Random-effects meta-analysis of the three case-control study periods – granular level workplace-related exposures, England, August–October 2020.

We explored the association between work-related exposures and infection using more granular level information on work activities (Fig. 3). There was evidence across all study periods that working in bars, restaurants and pubs was associated with elevated odds of being a case (pOR 2.87; aORs 3.52, 2.92, 2.41, for studies 1–3 respectively). Cases also had higher odds of working in a warehouse than controls (pOR 5.58; aOR range 1.72, 4.89, 24.06, for study periods 1–3 respectively). Working or attending secondary school settings was also associated with increased odds of being a COVID-19 case (pOR 2.58; aORs 1.52, 3.02, 2.98, for study periods 1–3 respectively). Working in primary school settings (pOR 1.43; aORs 0.43, 2.23, 2.58, for study periods 1–3 respectively) was associated with increased odds of being a COVID-19 case, especially in the second and third study periods. There was also evidence that working in hospital was linked with increased odds of COVID-19 (pOR 3.19; aORs 4.06, 2.29, 3.53, for study periods 1–3 respectively). Working in general practice surgeries (GPs) was associated with elevated odds of being a COVID-19 case (pOR 1.47; aORs 0.51, 2.17, 2.71, for study periods 1–3 respectively), with an increase in observed aORs over the three studies, along with evidence of heterogeneity (*I*^2^ = 69.4%, *P* = 0.04) (Fig. 3).

## Discussion

We observed important associations between certain occupations and SARS-CoV-2 infection. The associations between being a COVID-19 case and working in health and social care, hospitality, education or warehouse settings persisted over the three study periods. The findings highlight that infection control measures may have only a partial effect on reducing transmission in some workplace settings.

The findings on hospitality, primarily working in bars, pubs or restaurants, are supported by the accumulating evidence on the risk of COVID-19 associated with these settings from other countries [8, 10, 11, 20, 21]. Other studies have observed associations between exposure to hospitality venues and risk of infection that are similar to those observed in our study [12, 22]. These results are likely to reflect the ongoing risk in hospitality settings, especially among staff who work long hours and have multiple daily contacts, and may face multiple situations where adhering to social distancing is challenging [23].

The associations between certain settings and the odds of infection can only occur when there is an opportunity for the activities to take place. These opportunities are largely affected by NPIs, which in England have varied by geographical region and over time. It is therefore not surprising that, for example, the association between educational settings and increased odds of becoming a COVID-19 case was not observed in the first study period when most educational settings were closed. Educational settings have been associated with outbreaks [14, 15], and there is evidence of potential transmission events taking place in these settings [24]. However, the importance of educational settings in the context of community transmission of SARS-CoV-2 is still largely unclear [15].

Our findings on the associations between infection and working in manufacturing, construction and warehouse settings are consistent with the most commonly reported outbreak settings in European countries [8], Canada [13] and by other analytical studies [22]. It is plausible that sectors like construction may have different summer and winter working patterns with most of the work occurring indoors during the winter months, which may increase the opportunities for transmission in these settings. However, our study cannot account for this or other factors that may increase the opportunities for SARS-CoV-2 transmission outside workplaces. In general, use of shared facilities such as break rooms and kitchens, or high levels of car sharing or living in multioccupancy housing may create further opportunities for transmission, and while associated with workplace, they are not directly related to the occupation. Most of these factors, particularly those related to variation in individual behaviour, are still relatively poorly understood.

The findings regarding the association observed between working in healthcare (primarily associated with hospital exposure) and social care settings (primarily driven by exposure in care homes) and being a COVID-19 case were expected. These settings were closely associated with transmission in the first wave of the epidemic and, despite the appropriate use of personal protective equipment, healthcare workers experience high COVID-19 infection rates [7–9]. Our study identified that not only hospitals but also GP surgeries were associated with increased odds of infection.

Public health guidance and advice to reduce the risk of transmission in workplace settings have been published in England [25] and mitigation measures have been introduced. While compliance for these measures is generally high there is evidence that the effectiveness of these measures is only partial [16]. Furthermore, transmission of SARS-CoV-2 is driven by complex combinations of different environmental, social and behavioural factors that are challenging to fully quantify [2]. Hence, it is likely that, within the same occupational sector, there will be examples of good and poor practice in the implementation of measures to mitigate transmission, including considerable variation in individual behaviour. The risk of transmission is therefore likely to vary from setting to setting within a sector and the impact of workers' behaviour, including social interaction of staff during breaks, or before and after work, on transmission is not well understood. However, while it is not possible to determine the exact locations where transmission occurred, the associations we have observed are likely to reflect that it is more challenging to mitigate risk in some sectors compared to others.

Our study has a number of limitations. Across all three study periods, cases reported fewer exposures than controls. Cases are often first identified as contacts of other cases and it is possible that people modify their behaviour as a result. It is also possible that multiple biases affect the responses provided. One plausible explanation for this is that the cases going through the NHS T&T questionnaire may be experiencing high levels of questionnaire fatigue due to the length of the questionnaire. While the structure of the control survey (for exposures) was designed to be as identical to the case questionnaire as was practical, cases were required, as part of routine contact tracing, to provide considerable additional non-exposure information (e.g. symptoms, details and settings for close contacts). It is also possible that cases may feel inclined to adhere to socially desirable reporting, and not report activities they perceive would not be socially acceptable in the context of the pandemic. This differential misclassification of exposure most likely biased the effect measures towards the null, hence the results described are more likely to be underestimates rather than overestimates. There is also a potential for selection bias. As of the end of the study period three, altogether 630 309 cases were reached by contact tracing, with 82% of cases transferred to NHS T&T being reached by contact tracing [17]. This varied between 76.8% and 85.0% during our three study periods [17]. There is likely to be some selection bias among cases if those who did not engage in contact tracing differed from the rest in terms of their exposures. Testing procedures and pathways have also varied by time, geographical region and by workplace settings, with some workplace settings having higher level of routine testing than others, likely leading to differential capture of asymptomatic cases. The controls were sampled from a pool of volunteers to a commercial market research panel, which most likely introduced some selection bias for controls [26]. Furthermore, people registered and who chose to participate in the present studies might differ from those registered but who did not engage. The present analyses could only control for confounding by the exposure and demographic variables available, and residual confounding is likely to persist. However, the study did adjust for important, known confounding factors, including social deprivation, age, sex, ethnicity and geographical region. The observations presented in our study may be further confounded by other factors that were not part of the study including living conditions, transport to work or socio-economic factors not explained by small area social deprivation.

This study took place in the context of COVID-19 pandemic response when there was an urgent need to provide timely evidence on the risks associated with community exposures. The findings from these sampling periods provided evidence which together with the wider pool of evidence was used to inform risk assessments, policies and targeted public health action in the operational context of the pandemic response. The study allowed rapid, concurrent data collection from both cases and controls, providing epidemiological insights with consistent estimates of associations reported over three study periods. To the best of our knowledge, this is the only case-control study on community risk factors for COVID-19 in England. As such, this study provides valuable insights into the risks associated with community settings and activities, and the findings are likely to be transferrable to other similar settings.

We conclude that several workplaces were associated with increased odds of being a COVID-19 case, primarily those in health and social care, hospitality, warehouse and educational settings. The results are aligned with the existing literature. While it was not possible to determine whether transmission occurs at workplaces or outside the workplace, for example, during commuting, our findings provide further evidence that certain sectors have been more affected, highlighting that the mitigation measures in these settings may only be partial. While the study has limitations, these are likely to result in underestimation, rather than overestimations of the associations. Therefore, our recommendation is that further mitigation measures are considered in these sectors to improve the control of COVID-19 in these settings.

## Data Availability

The data that support these studies were collected as part of a public health response, are considered sensitive and not made publicly available. Reasonable requests for access to anonymised data will be considered by the authors on request.
